# Importance of mitochondrial calcium uniporter in high glucose–induced endothelial cell dysfunction

**DOI:** 10.1177/1479164117723270

**Published:** 2017-08-04

**Authors:** Wei Chen, Jie Yang, Shuhua Chen, Hong Xiang, Hengdao Liu, Dan Lin, Shaoli Zhao, Hui Peng, Pan Chen, Alex F Chen, Hongwei Lu

**Affiliations:** 1 Center for Experimental Medical Research, The Third Xiangya Hospital of Central South University, Changsha, Hunan, P.R. China; 2 Department of Cardiology, The Third Xiangya Hospital of Central South University, Changsha, Hunan, P.R. China; 3 Department of Biochemistry, School of Life Sciences of Central South University, Changsha, Hunan, P.R. China; 4 Qingdao Municipal Center for Disease Control and Prevention, Qingdao, Shandong, P.R. China; 5 Department of Surgery, University of Pittsburgh School of Medicine, Pittsburgh, PA, USA

**Keywords:** High glucose, endothelial cell, mitochondrial calcium uniporter, mitochondrial calcium uniporter regulator 1

## Abstract

**Objective:**

Mitochondrial Ca^2+^ overload is implicated in hyperglycaemia-induced endothelial cell dysfunction, but the key molecular events responsible remain unclear. We examined the involvement of mitochondrial calcium uniporter, which mediates mitochondrial Ca^2+^ uptake, in endothelial cell dysfunction resulting from high-glucose treatment.

**Methods:**

Human umbilical vein endothelial cells were exposed to various glucose concentrations and to high glucose (30 mM) following mitochondrial calcium uniporter inhibition or activation with ruthenium red and spermine, respectively. Subsequently, mitochondrial calcium uniporter and mitochondrial calcium uniporter regulator 1 messenger RNA and protein expression was measured by real-time polymerase chain reaction and western blotting. Ca^2+^ concentrations were analysed by laser confocal microscopy, and cytoplasmic and mitochondrial oxidative stress was detected using 2′,7′-dichlorofluorescein diacetate and MitoSOX Red, respectively. Apoptosis was assessed by annexin V-fluorescein isothiocyanate/propidium iodide staining, and a wound-healing assay was performed using an in vitro model.

**Results:**

High glucose markedly upregulated mitochondrial calcium uniporter and mitochondrial calcium uniporter regulator 1 messenger RNA expression, as well as protein production, in a dose- and time-dependent manner with a maximum effect demonstrated at 72 h and 30 mM glucose concentration. Moreover, high-glucose treatment significantly raised both mitochondrial and cytoplasmic Ca^2+^ and reactive oxygen species levels, increased apoptosis and compromised wound healing (all *p* < 0.05). These effects were enhanced by spermine and completely negated by ruthenium red, which are known to activate and inhibit mitochondrial calcium uniporter, respectively.

**Conclusion:**

Mitochondrial calcium uniporter plays an important role in hyperglycaemia-induced endothelial cell dysfunction and may constitute a therapeutic target to reduce vascular complications in diabetes.

## Introduction

Endothelial cells (ECs) play a crucial role in maintaining vascular homeostasis,^1^ and dysfunction of the vascular endothelium is associated with the pathogenesis of cardiovascular diseases.^2^ Moreover, compelling evidence has implicated dysfunctional ECs in the development of diabetic vascular diseases,^3,4^ the significance of which is underscored by the World Health Organization’s estimate that there are currently 422 million patients with diabetes worldwide.^5^ The classical in vitro model of diabetes development comprises the induction of endothelial dysfunction by high glucose (HG) levels. Therefore, understanding the molecular mechanism by which vascular EC dysfunction arises under HG conditions may be useful for both the prevention and treatment of diabetic vascular complications.

Many reports have shown that calcium is an important regulator of endothelial function in diabetes,^6–8^ and mitochondria constitute one of the largest intracellular calcium pools. However, the key signalling events linking hyperglycaemia with mitochondrial Ca^2+^ concentration in ECs are not known. Mitochondrial calcium uniporter (MCU) is the pore-forming subunit of the Ca^2+^ uniporter ion channel located on the mitochondrial inner membrane that mediates Ca^2+^ uptake into the matrix, regulating cytoplasmic Ca^2+^ signalling.^9–11^ MCU is essential for glucose-induced increases in adenosine triphosphate (ATP) concentration in pancreatic β-cells,^12^ and its knockdown reduces agonist- and depolarization-induced mitochondrial Ca^2+^ sequestration, ATP production, and d-glucose-stimulated insulin secretion.^13^ However, the role of MCU in hyperglycaemia-induced EC dysfunction has not been explored.

We propose that MCU is the critical protein responsible for mitochondrial Ca^2+^ overload in EC dysfunction caused by hyperglycaemia. Mitochondrial calcium uniporter regulator 1 (MCUR1) is also essential for the formation of MCU complex and may regulate its function, enabling mitochondrial Ca^2+^ uptake and the maintenance of normal cellular bioenergetics.^14^ Reactive oxygen species (ROS) overproduction, increased apoptosis and compromised wound-healing ability contribute to the development of pathological conditions associated with endothelial dysfunction.^15,16^ Thus, in this study, we used human umbilical vein endothelial cells (HUVECs) to investigate the involvement of MCU and MCUR1 in hyperglycaemia-induced mitochondrial Ca^2+^ overload, ROS overproduction, apoptosis and decreased wound-healing ability.

## Materials and methods

### Cell culture and treatment

HUVECs were purchased from the American Type Culture Collection (Manassas, VA, USA) and were cultured in low-glucose Dulbecco’s Modified Eagle’s medium (DMEM) containing 10% foetal bovine serum, 100 U/L penicillin and 100 U/L streptomycin at 37°C in 5% CO_2_. Cells of passages 3–7 were used for experiments. As described in previous studies, the cells were treated with different concentrations of glucose (2, 5.5, 10, 20 and 30 mM) for 72 h and exposed to HG (30 mM) for different lengths of time (0, 12, 24, 48 and 72 h).^17,18^ Treatment with 5.5 mM glucose was considered the ‘normal glucose’ (NG) condition. Once the optimal glucose exposure time and concentration had been established, experiments involving 30-min pre-treatment with the MCU inhibitor ruthenium red (RR; 0.2 mM) (Sigma, St. Louis, MO, USA) or MCU activator spermine (sper; 2 mM) (Sigma) were carried out. As an osmotic control, treatment with 24.5 mM mannitol (Mnt) and 5.5 mM glucose was used.

### Real-time polymerase chain reaction

Total RNA was isolated from HUVECs using Total RNA Kit I (Omega Bio-tek, Norcross, GA, USA) and reverse transcribed into complementary DNA (cDNA) with a ReverTra Ace qPCR RT Master Mix with gDNA Remover kit (Toyobo, Osaka, Japan). In all, 50-ng samples of this cDNA were then subjected to real-time polymerase chain reaction (PCR) on a Mastercycler nexus X2 PCR System (Eppendorf, Hamburg, Germany) using SYBR Green I and TaqMan probes and primers targeting *MCU* (forward: TTCACCTCTTCTGGGAGCAG; reverse: TCCAGGATCTTGACCAATGCT), *MCUR1* (forward: ATTCCTGGGACATCATGGAG; reverse: TGTCTGTCTCTGGCTTCTGG) and *β-actin* (forward: GAGACCTTCAACACCCCAG; reverse: TCAGGTCCCGGCCAGCCA). Values for each unknown were determined by the 2^−ΔΔCt^ method, using β-actin as a reference sequence and standard curves derived from samples of known RNA quantities.

### Western blotting

Total protein extracts (30 µg) were electrophoresed on a 10% sodium dodecyl sulphate–polyacrylamide gel. The separated proteins were transferred electrophoretically to a polyvinylidene difluoride (PVDF) membrane, which was blocked with a 5% skimmed milk solution for 2 h at 25°C and incubated with primary antibodies against MCU and MCUR1 (Abcam, Cambridge, UK; 1:500) overnight at 4°C. After washing, the blots were incubated with a goat anti-rabbit IgG horseradish peroxidase (HRP)-conjugated secondary antibody (Abcam; 1:3000) for 2 h at 25°C. Antigen–antibody complexes were visualized using ECL Western Blotting Detection Reagents and an ImageQuant 350 instrument (GE Healthcare, Marlborough, MA, USA). Once stripped, the membranes were further probed with an antibody against β-actin as a loading control. Greyscale values were calculated from blots using ImageJ software.

### Measurement of [Ca^2+^]mito and [Ca^2+^]cyt by laser confocal microscopy

Changes in [Ca^2+^]mito and [Ca^2+^]cyt were detected by incubating HUVECs for 30 min at 37°C in the dark with the Ca^2+^ indicators Rhod-2 AM (Molecular Probes, Eugene, OR, USA) and Fluo-3 AM (Beyotime, Jiangsu, China) at final concentrations of 2 and 5 µmol/L, respectively. After incubation, the cells were washed twice with phosphate-buffered saline (PBS) and analysed by capturing fluorescence images using a confocal microscope equipped with an argon–krypton laser. Fluorescence deriving from Rhod-2 AM and Fluo-3 AM was assessed with 559/578 and 488/515 nm excitation/emission filters, respectively.

### Intracellular ROS measurement

Intracellular ROS levels were measured using the 2′,7′-dichlorofluorescein diacetate (DCF-DA) assay. After treatment, HUVECs were stained with 10 µM DCF-DA (Beyotime, Haimen, China) for 30 min at 37°C in the dark, before being washed twice with PBS. ROS production was then assessed using a fluorescent microscope (Nikon, Tokyo, Japan) with a fluorescein isothiocyanate (FITC) green filter set (485/535 nm excitation/emission). Fluorescence intensities were determined using ImageJ software.

### Measurement of mitochondrial $O_{2}^{-}$ levels

Changes in mitochondrial $O_{2}^{-}$ levels were measured using MitoSOX Red (Yeasen, Shanghai, China), which targets mitochondria and fluoresces when oxidized by $O_{2}^{-}$. Subcultured HUVECs were loaded with MitoSOX Red, which was dissolved in dimethyl sulphoxide (DMSO) and used at a final concentration of 5 µmol/L, for 30 min at 37°C in the dark, and washed twice with PBS. Mitochondrial $O_{2}^{-}$ levels were then assessed using a fluorescence microscope with 510 and 580 nm excitation and emission filters, respectively, and fluorescence intensities were determined using ImageJ software.

### Flow cytometric analysis of EC apoptosis

To quantify HUVEC viability, cells were stained with both annexin V and propidium iodide (PI) using an Annexin V-FITC Apoptosis Detection Kit (BD Biosciences, Franklin Lakes, NJ, USA) according to the manufacturer’s instructions. The proportion of apoptotic cells was determined using a FACSAria instrument (BD Biosciences).

### Wound-healing assay

HUVECs were plated on six-well plates and grown overnight to near confluence. A cell-free area was made in each well by scraping a 10 µL pipette tip across the cells, and culture was then continued. Images were taken 0, 18 and 36 h after creating the wounds, and cells migrating into the cell-free space were quantified.

### Statistical analysis

Data are expressed as means ± SEM. Comparisons between more than two groups were performed using one-way analysis of variances (ANOVAs) and a post hoc Tukey test. Comparisons between two groups were performed using unpaired *t*-test. *p*-values <0.05 were considered statistically significant. When individual studies are demonstrated, there are representative of at least three independent studies.

## Results

### Effects of glucose on MCU and MCUR1 expression in HUVECs

**Figure 1.**
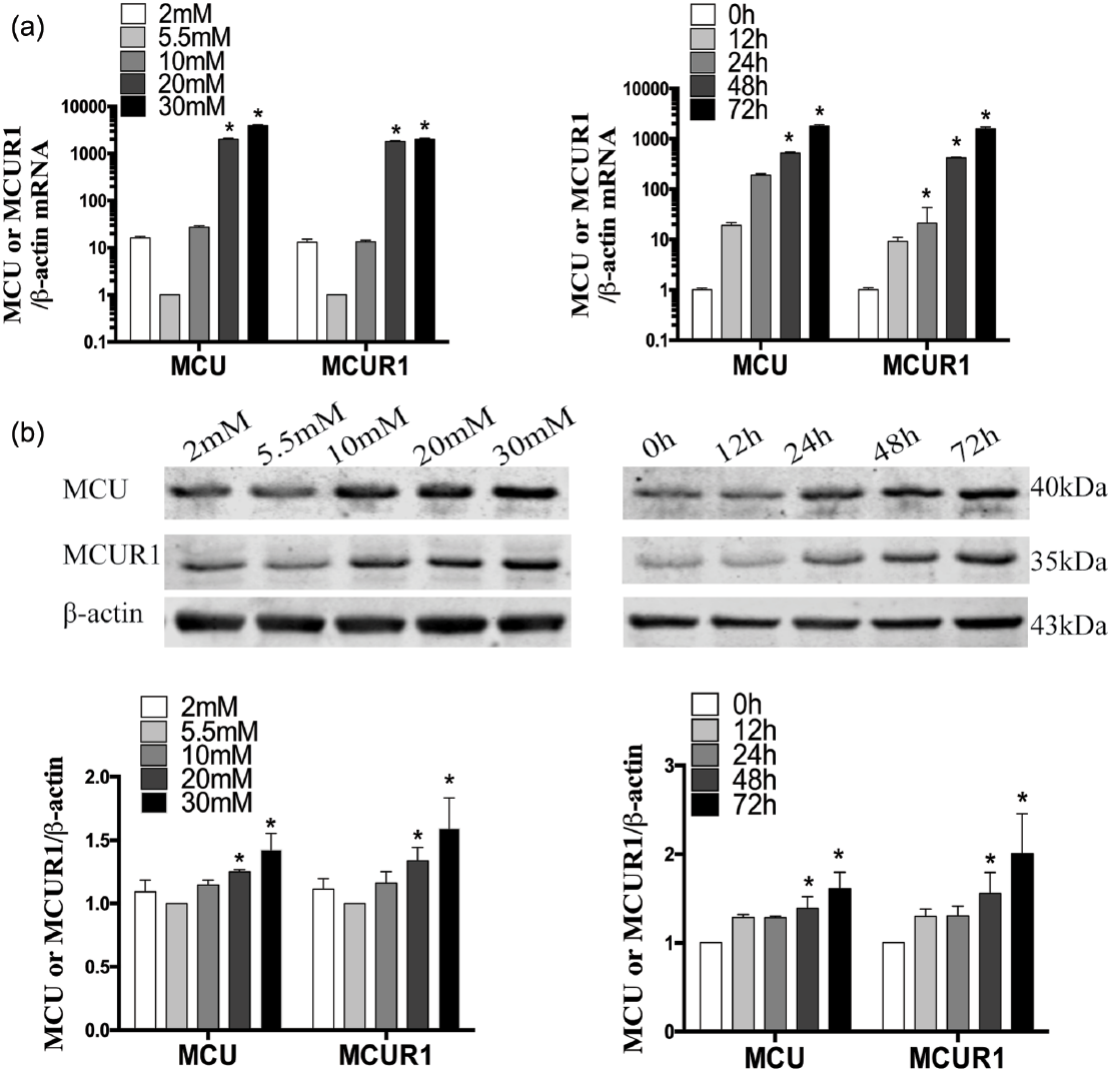
MCU and MCUR1 mRNA and protein expression in HUVECs cultured in the presence of HG. (a) Cells were treated with different glucose concentrations (2, 5.5, 10, 20 and 30 mM) for 72 h, and HG (30 mM) for various periods (0, 12, 24, 48 and 72 h). (b) MCU, MCUR1 and β-actin (the latter as a loading control) mRNA and protein levels were evaluated by real-time PCR and western blotting, respectively. Data are expressed as the means ± SEM of three independent experiments. Results were analysed by unpaired *t*-test. *p < 0.05 versus the NG group.

As shown in Figure 1, the lowest glucose concentration used (2 mM) slightly elevated MCU and MCUR1 messenger RNA (mRNA) and protein expression in HUVECs, although this effect was not statistically significant. However, HG significantly increased both the mRNA and protein levels of MCU and MCUR1 in these cells (*p* < 0.05) in a time-dependent manner. These levels were highest using 30 mM glucose for 72 h (*p* < 0.05).

### Effects of glucose on calcium and ROS levels in HUVECs

**Figure 2.**
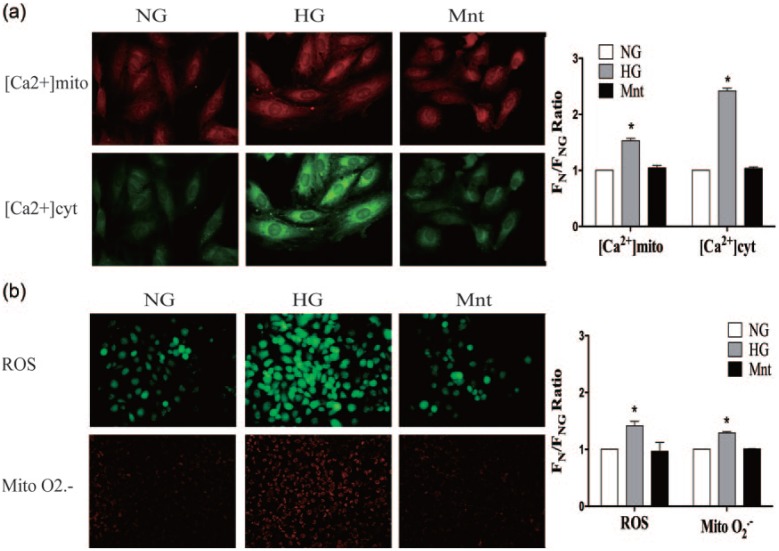
Cytoplasmic and mitochondrial calcium and oxidative stress levels in HUVECs cultured in the presence of HG. (a) Cells were treated with NG, HG or Mnt for 72 h, and calcium concentrations in the mitochondria ([Ca^2+^]mito) and cytoplasm ([Ca^2+^]cyt) were determined by laser confocal microscopy following exposure to the Ca^2+^ indicators Rhod-2 AM and Fluo-3 AM. (b) Total ROS and mitochondrial $O_{2}^{-}$ levels were quantified by fluorescence microscopy following DCF-DA and MitoSOX Red staining, respectively. Data are expressed as the means ± SEM of three independent experiments. Results were analysed by one-way ANOVA followed by Tukey’s post hoc test. *p < 0.05 versus the NG group.

[Ca^2+^]mito and [Ca^2+^]cyt were significantly elevated in cells of the HG group (*p* < 0.05) (Figure 2(a)). Moreover, total ROS and mitochondrial $O_{2}^{-}$ levels in HUVECs treated with HG were remarkably higher than in cells exposed to the NG and Mnt condition (*p* < 0.05) (Figure 2(b)). There was no significant difference in [Ca^2+^]mito, [Ca^2+^]cyt, total ROS and mitochondrial $O_{2}^{-}$ levels between NG and Mnt group (*p* > 0.05) (Figure 2).Thus, HG not only increased the expression of MCU and MCUR1 but also resulted in heightened calcium and oxidative stress levels in the cytoplasm and mitochondria of HUVECs.

### Effects of glucose on HUVEC apoptosis and wound healing

**Figure 3.**
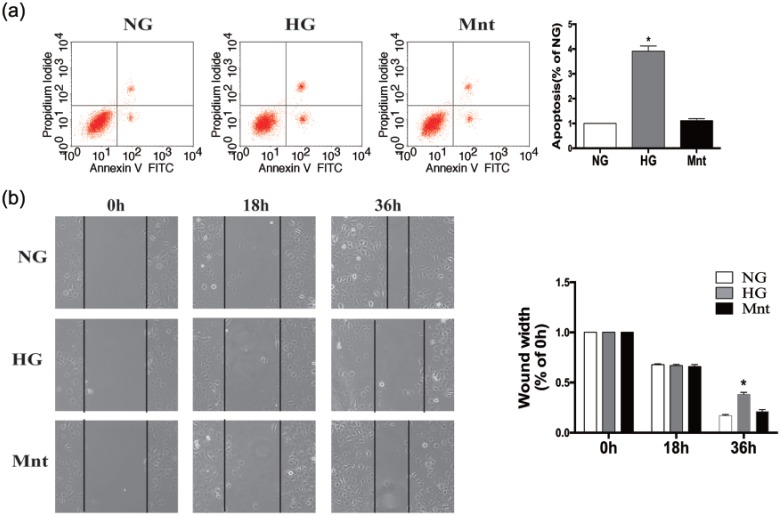
Effects of HG on apoptosis of wound-healing ability of HUVECs. (a) Cells were treated with NG, HG or Mnt for 72 h, before being stained with both annexin V and PI and subjected to flow cytometric analysis. (b) Degree of wound closure after 0, 18 and 36 h for each treatment group was quantified using a microscope. Results are expressed as means ± SEM (*n* = 3). Results were analysed by one-way ANOVA followed by Tukey’s post hoc test. *p < 0.05 versus the NG group.

As shown in Figure 3(a), HUVEC apoptosis increased significantly following treatment with HG for 72 h. In addition, the degree of wound closure in the HG group was significantly lower than that in the NG and Mnt groups (Figure 3(b)). There was no significant difference in apoptosis and wound healing between NG and Mnt groups (*p* > 0.05) (Figure 3). These results suggest that HG exposure can impair ECs function.

### Role of MCU in hyperglycaemia induced increase in HUVEC calcium and ROS levels

**Figure 4.**
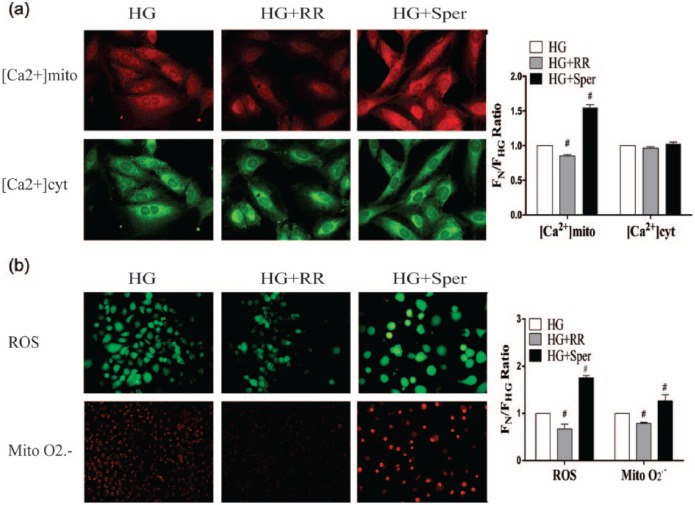
MCU inhibition negates HG-induced oxidative stress and mitochondrial Ca^2+^ overload in HUVECs. HUVECs were pre-treated for 30 min with ruthenium red (RR) or spermine (sper), which, respectively, inhibits and activates MCU, and exposed to HG for 72 h. (a) [Ca^2+^]mito, [Ca^2+^]cyt, and (b) levels of total ROS and mitochondrial $O_{2}^{-}$ were then quantified. Data are expressed as the means ± SEM from three independent experiments. Results were analysed by unpaired *t*-test. #*p* < 0.05 versus the HG group.

To explore the role of MCU in calcium and ROS changes in ECs during hyperglycaemia, HUVECs were pre-treated for 30 min with RR or sper, which inhibits and activates MCU, respectively, and exposed to HG for 72 h. [Ca^2+^]mito was significantly reduced in the HG + RR group and elevated in the HG + sper group (*p* < 0.05) (Figure 4(a)); however, [Ca^2+^]cyt did not significantly differ following these treatments, compared to the HG group. As shown in Figure 4(b), in comparison with HUVECs treated with HG only, total ROS and mitochondrial $O_{2}^{-}$ levels were significantly lower in cells treated HG + RR, and significantly higher in those exposed to HG + sper. These results demonstrate that MCU plays a crucial role in hyperglycaemia-induced mitochondrial calcium overload and oxidative stress in HUVECs.

### Roles of MCU in hyperglycaemia induced HUVEC apoptosis and compromised wound-healing ability

**Figure 5.**
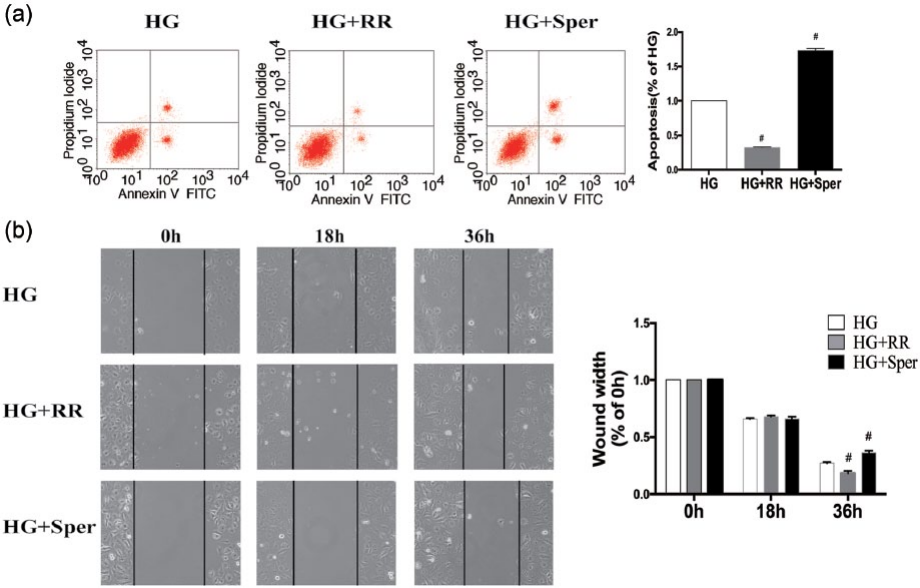
MCU inhibition negates hyperglycaemia-induced HUVEC dysfunction. HUVECs were pre-treated for 30 min with ruthenium red (RR) or spermine (sper) before being subjected to HG treatment for 72 h. (a) The incidence of apoptosis and (b) degree of wound closure were quantified. Results are expressed as means ± SEM (*n* = 3). Results were analysed by unpaired *t*-test. #*p* < 0.05 versus the HG group.

As shown in Figure 5(a), compared to the HG group, apoptosis was less frequent among HUVECs treated with HG + RR and more frequent among those administered HG + sper. Also in comparison with the HG-treated group, gap closure was significantly accelerated and retarded by HG + RR and HG + sper treatment, respectively. These data indicate that MCU exerts an important effect on hyperglycaemia-induced EC apoptosis and wound healing.

## Discussion

Mitochondrial Ca^2+^ overload has been implicated as a critical mediator of cellular dysfunction under hyperglycaemic conditions,^7^ but the key signalling events linking hyperglycaemia with increased mitochondrial Ca^2+^ have so far remained elusive. Here, we demonstrate for the first time that hyperglycaemia-induced mitochondrial Ca^2+^ overload depends on MCU activation, leading to increased mitochondrial ROS levels and HUVEC dysfunction.

First, our results showed that HG increased HUVEC MCU and MCUR1 expression at transcriptional and translational levels in a dose- and time-dependent manner. Moreover, HG resulted in elevated Ca^2+^ and ROS levels in these cells, increased their rate of apoptosis and compromised their wound-healing ability. Hyperglycaemia was thus found to induce EC dysfunction, as reported in previous studies.^16,19^ Furthermore, our data suggest that MCU and MCUR1 are upregulated in ECs after HG exposure, providing evidence for the role of the MCU complex in diabetic vascular diseases.

MCU is a mitochondrial inner membrane uniporter that mediates mitochondrial uptake of Ca^2+^ and controls its concentration within this organelle.^20^ Previous studies have shown that excessive mitochondrial Ca^2+^ uptake has deleterious effects, including ROS overproduction,^21^ sensitization to apoptosis,^22^ and activation of the permeability transition pore and subsequent cell death pathways.^23,24^ Our results demonstrate that hyperglycaemia results in increased MCU expression and impaired cell function. However, little is known of the significance of MCU in HG-induced EC dysfunction. In order to address this gap in our knowledge, we exposed HUVECs to an inhibitor or an activator of MCU prior to HG treatment. The Ca^2+^ overload, heightened oxidative stress, increased apoptosis rate and decreased wound-healing ability induced by hyperglycaemia, and these effects were negated by MCU inhibition. Conversely, the MCU activator spermine was found to exacerbate HG-induced EC dysfunction. These results support the conclusion that MCU is clearly an important mediator of the detrimental effects of HG on EC function.

Consistent with previous results,^25,26^ we found MCU to be associated with oxidative stress-induced apoptosis. Knocking down endogenous MCU has been shown to decrease mitochondrial Ca^2+^ uptake and attenuate the death of HeLa cells and primary cerebellar granule neurons resulting from oxidative stress.^25^ In addition, Sripetchwandee et al.^26^ established that iron overload could result in cardiac mitochondrial dysfunction, including increased ROS production, membrane depolarization and swelling, and showed that these effects could be prevented by MCU blockers. MCU is also necessary for store-operated Ca^2+^ entry-dependent breast cancer cell migration.^27^ Our results confirm an essential role for MCU in the Ca^2+^ overload that leads to deleterious consequences for ECs. However, further investigation is required to identify the signalling pathways downstream of MCU involved in EC dysfunction.

To conclude, our study suggests that hyperglycaemia results in enhanced expression of MCU and MCUR1, leading to mitochondrial Ca^2+^ overload. Subsequent excessive generation of ROS due to heightened mitochondrial activity may then eventually cause endothelial dysfunction. We propose that MCU plays an important role in diabetic vascular diseases. Therefore, targeting MCU or up- or down-stream factors may constitute a novel therapeutic approach for patients with diabetes and vascular complications.

**Key messages**

- High-glucose conditions result in endothelial cell (EC) dysfunction.
- Ca^2+^ overload may be responsible for the effects of hyperglycaemia on ECs.
- Mitochondrial calcium uniporter (MCU) and mitochondrial calcium uniporter regulator 1 (MCUR1) are upregulated under high-glucose conditions in ECs.
- Inhibiting and activating MCU, respectively, negates and exacerbates high glucose–induced EC dysfunction.
- MCU may be a key factor and drug target in diabetic vascular injury.
